# The influence of rapid eye movement sleep deprivation on nociceptive transmission and the duration of facial allodynia in rats: a behavioral and Fos immunohistochemical study

**DOI:** 10.1186/s10194-019-0977-0

**Published:** 2019-03-01

**Authors:** Seong Hoon Kim, Ju Yeon Park, Hae Eun Shin, Si baek Lee, Dong Woo Ryu, Tae Won Kim, Jeong Wook Park

**Affiliations:** 1 Department of Neurology, The Catholic University of Korea, College of Medicine, Uijeongbu St Mary’s Hospital, 65-1 Geumo-dong, Uijeongbu, Gyeonggi Do South Korea; 2 Department of Neurology, The Catholic University of Korea, College of Medicine, Incheon St Mary’s Hospital, Incheon, South Korea

**Keywords:** REM sleep deprivation, Headache, Facial allodynia, Capsaicin

## Abstract

**Background:**

Disrupted sleep is associated with a reciprocal influence on headaches and is one of the contributing factors in the process of chronicity. The goal of the present study was to investigate the influence of sleep on headaches using animal rapid eye movement (REM) sleep deprivation and supradural capsaicin infusion models.

**Method:**

Sprague-Dawley rats underwent REM sleep deprivation (REMSD) for 96 h. The sensory threshold to mechanical stimuli, assessed by the von Frey monofilament test, was measured during the REMSD period. Additionally, the Fos protein expression level was measured in the trigeminocervical complex, periaqueductal gray, and hypothalamus. Following supradural infusion of capsaicin, we evaluated the duration of facial allodynia for 28 days after REMSD.

**Results:**

After REMSD, the sensory threshold to mechanical stimuli was significantly decreased (*p* < 0.01) and Fos-positivity in the posterior (*p* = 0.010) and dorsomedial hypothalamus (*p* = 0.024), ventrolateral periaqueductal gray (*p* = 0.016), and superficial layer of the trigeminocervical complex (*p* = 0.019) were significantly increased. The duration of facial allodynia induced by supradural capsaicin infusion was significantly longer in the REM sleep deprivation and capsaicin infusion group (Day 10 PSD vs. Day 25 PSD).

**Conclusion:**

The present study demonstrates that REM sleep deprivation increased nociceptive transmission from trigeminal nerve endings. Furthermore, it suggests that sleep deprivation may contribute to the chronicity of facial allodynia.

## Introduction

Although the physiological role of sleep is still not fully understood, it is thought to be related to metabolic, cognitive, and homeostatic regulation [1, 2]. Sleep deprivation is common even in healthy individuals and can bring about neurobiological changes [1, 3]. The generally accepted perspective in the scientific community is that sleep deprivation lowers the pain threshold and enhances pain transmission in various chronic pain diseases [4–6]. Recent work has shown that sleep problems are a stronger predictor of pain, and they increase the risk of chronic pain [7, 8].

Sleep deprivation reduces the central inhibitory influence on pain throughout the endogenous enkephalinergic, orexinergic, and serotonergic pathways [9–13]. The hypothalamus and periaqueductal gray have been implicated in descending modulation of pain through their connections with the trigeminal nucleus caudalis; they provide a putative mechanism for the circadian regulation of sleep–wake cycles [14, 15]. Recent research has demonstrated that sleep deprivation increases nociceptive responses by disrupting descending pain modulation mechanisms or adenosine and dopaminergic receptors in forebrain nuclei [8, 15].

Chronic headaches sometimes present difficulties related to clinical management and can have a profound impact on a patient’s life [16]. Sleep problems are known to be one of the major contributing factors to the development of chronic headaches [17]. Facial allodynia, which occurs in a considerable number of patients, is known to be associated with central sensitization and treatment resistance [18]. The periorbital area is innervated from the ophthalmic division of the trigeminal nerve and connected to trigeminocervical complexes, and this is important in the development of headaches [19, 20]. The presence of allodynia is also known to be related to the onset of chronic headaches, especially migraines [21]. Supradural inflammatory infusion in awake and freely moving rats has been proposed as a methodological tool for facial allodynia associated with migraines based on animal research [22]. Repeated infusion of an inflammatory stimulant induced hypersensitivity to mechanical stimuli in the face; this hypersensitivity lasted for at least three weeks and has been suggested as an animal model of chronic headaches [23].

We hypothesized that sleep deprivation aggravates nociceptive transmission from the trigeminal ophthalmic division and contributes to chronicity. We measured changes in sensitivity to mechanical stimuli during 96 h of rapid eye movement (REM) sleep deprivation and histopathological changes in structures related to nociceptive transmission. To focus on chronicity, we applied the supradural capsaicin infusion model and measured the duration of facial allodynia for 28 days after cessation of REM sleep deprivation.

## Methods

### Animal preparation

Experiments were conducted following an ethical review by the Institutional Animal Care and Use Committee of The Catholic University of Korea (approval no. UJA 2014-08A, UJA 2014-12A). Male Sprague-Dawley rats (190–220 g) were placed in standardized plastic cages with sawdust bedding in a temperature-controlled room (22 °C ± 2 °C) with a 12-h light–dark cycle. All procedures took place in a quiet room during the light phase, between 10:00 and 13:00.

### Sleep deprivation paradigm

We used the single-platform technique to induce REM sleep deprivation. The animals were individually maintained for 96 h in water chambers measuring 22.0 cm long, 22.0 cm wide, and 35.0 cm high. Within the chambers, the rats were placed onto a 6.5-cm-diameter platform atop a 15-cm-high pedestal that was immersed in water up to 1.0 cm below the platform’s upper surface. Whenever rats on the platforms lapsed into REM sleep, the loss of muscle tone caused them to make facial contact with the water and they would subsequently awaken [24]. Control rats were kept in a similar chamber filled with sawdust bedding instead of water.

All rats were habituated to the experimental environment for one hour per day for two consecutive days preceding the onset of the study. The purpose of this routine was to train each animal to balance on the platform to avoid excessive falling into the water during the sleep deprivation period. Body weights and the amount of food eaten during the previous day were estimated daily. Stainless steel clamps held the food container in place within easy access of the platform; drinking water was provided with an overhead bottle.

### Supradural capsaicin infusion

A surgical procedure involving placement of a supradural indwelling catheter was performed according to a protocol previously published by Wieseler et al. [22]. Briefly, the rats were anesthetized using isoflurane and placed in a stereotaxic apparatus (Kopf Instruments, Tujunga, CA, USA). All surgical tools were sterilized before use. After shaving the surgical site, a 1–2-cm skin incision was made to expose the bregma while trying to minimize bleeding. A dental drill was used with a Dremel #107 engraving cutter bit to bore two 2-mm-wide, 10-mm-long troughs into the skull. These two troughs were positioned 3–4 mm parallel to the midsagittal suture. Troughs were drilled such that the dura was exposed 1 mm caudal to bregma, while trying not to penetrate the dura. After the bilateral troughs were drilled, polyethylene catheters (PE-10; BD, Franklin Lakes, NJ, USA) were carefully inserted horizontally along the troughs. The catheters were immobilized with Superglue (3 M, Maplewood, MN, USA) and allowed to dry. The external seal of each catheter was then clipped, and the catheters were flushed with 5 μl of sterile saline, with care being taken not to introduce any air or occlusion into the catheter. The scalp wound was sealed with 9-mm stainless steel wound clips. Subsequently, the rats were individually housed once ambulatory. As an attempt to prevent clogging, 2 μm of heparin was flushed through the catheter at 72 h after surgery.

Four days after supradural catheter insertion, capsaicin 100 nM (1% ethanol in saline) was injected at a total amount of 16 μl (capsaicin: 10 μl, sterile saline in catheter: 6 μl) serially over four minutes using an infusion pump (Harvard Apparatus, Holliston, MA, USA) while the rat was freely moving. The injector remained connected to the catheter for one-minute following infusion to allow diffusion of the solution. The same procedure was repeated after two hours. Catheter placement was verified at the completion of all experiments.

### Von Frey monofilament test

The sensory threshold to mechanical stimuli at the facial area was verified by the von Frey monofilament (VFMF) threshold test. All testing was performed with blinding with respect to group assignment. For the VFMF test, each rat was placed in an atraumatic plastic tube restraint, into which the animal entered uncoaxed. Animals were placed in the testing apparatus for acclimation through a training period. The sensory thresholds were determined by applying the VFMF apparatus (North Coast Medical, Inc., Gilroy, CA, USA) to the face over the periorbital region on both sides. The monofilaments were presented in either a sequential ascending or descending order for determining the threshold of response, as published previously [25, 26]. A positive response to the VFMF test was noted when the rat moved its head to avoid stimulus or struck its face with its ipsilateral forepaw. The 66% probability threshold of a positive response, defined as a positive response to two of three trials during the VFMF test, was determined.

### Study groups

All male Sprague-Dawley rats were randomly assigned to four groups using the random function (=RAND) in Microsoft Excel (Microsoft Corp., Redmond, WA, USA), as follows:

- Ad libitum sleep group (NonSD): animals stayed in the chamber with sawdust bedding for 96 h (*n* = 6)
- REM sleep deprivation group (REMSD): animals stayed in the water chamber for 96 h (*n* = 6)
- Ad libitum sleep and capsaicin infusion group (NonSD-Capsaicin): animals received injections of capsaicin and were kept in a chamber with sawdust bedding for 96 h (*n* = 8)
- REM sleep deprivation and capsaicin infusion group (REMSD-Capsaicin): animals received injections of capsaicin and were kept in the water chamber for 96 h (*n* = 6)

### The course of the study

In the first part of the study, the effects of REM sleep deprivation on sensory threshold to mechanical stimuli and neuronal activation were assessed in the NonSD group and the REMSD group. In these experiments, the VFMF thresholds were assessed every day during sleep deprivation. After sleep deprivation, the brains of each group were analyzed by immunohistochemical staining.

In the second part of the study, the effects of REM sleep deprivation on facial allodynia induced by supradural capsaicin infusion were assessed in the NonSD-Capsaicin group and the REMSD-Capsaicin group. Animals involved in the first part of the study did not participate in any sessions of the second part of the study. In this part, the VFMF threshold test was performed for 28 days after REMSD.

### Tissue preparation

The REMSD and NonSD groups were sacrificed by injection with 0.9% saline (300 mL), followed by 4% paraformaldehyde (300 mL) in 0.1 M phosphate-buffered saline (PBS; pH: 7.4) via the ascending aorta. The brain and cervical spinal cord were stored overnight in the same fixative and then placed in a cryoprotectant solution (30% sucrose in 0.1 M PBS) for 48 h. After serial cutting using a freezing cryostat, sections (30 μm) that spanned the hypothalamus, periaqueductal gray (PAG), trigeminocervical complex (composed of the trigeminal nucleus caudalis and cervical spinal cord levels C1, C2, and C3; TCC) were collected in PBS. Tissue sections were processed as free-floating sections. Sections were placed in and rinsed with 0.1 M PBS in 24-well plates and then incubated in 3% H_2_O_2_ in 50% ethanol (Merck, Darmstadt, Germany) for 15 min. Each section was incubated in a blocking solution (10% normal goat serum in 0.1 M PBS), followed by incubation overnight with a rabbit primary anti-Fos antibody (1,5000; ab99515; Abcam, Cambridge, UK) diluted in 4% BSA, 2% normal goat serum, and 0.2% Triton X-100 in PBS. The next morning, sections were incubated in a secondary antibody (a biotinylated goat anti-rabbit antibody; Vector, Burlingame, CA, USA) diluted at 1:500 in 4% BSA, 2% normal goat serum, and 0.2% Triton X-100 in PBS for 20 min, followed by further washes in PBS. Sections were then incubated with ExtrAvidin–peroxidase (Sigma-Aldrich, St Louis, MO, USA) for two hours. Finally, the sections were incubated with 3,3′-diaminobenzidine tetrahydrochloride dihydrate containing nickel (Vector, Burlingame, CA, USA) before being washed and mounted on slides, air-dried, dehydrated, and mounted in a distyrene plasticizer xylene medium under a coverslip (Paul Marienfeld GmbH & Co. KG, Lauda-Königshofen, Germany). The omission of either the primary or the secondary antibody abolished the immunohistochemical staining completely, indicating specificity. Fos immunoreactivity was distinguishable by cellular location. That is, the nucleus was always identified by an intense dark brown-to-black color, indicating the presence of the Fos protein. All sections were examined under an Olympus Universal microscope equipped with a digital camera (Olympus, Shinjuku, Tokyo, Japan) by an investigator blinded to the animal group being assessed.

Cytoarchitecturally identified regions of the hypothalamus, PAG, and TCC of hemisections were used for confirmation of Fos-positive cells. Localization of the hypothalamus, PAG, and TCC was done with reference to the obex according to coordinates provided by Paxinos and Franklin [27]. For the hypothalamus, four brain regions were selected for quantitative analysis: the posterior hypothalamus (P-HT), dorsomedial hypothalamus (DM-HT), ventromedial hypothalamus, and lateral hypothalamus. Three brain regions were selected for quantitative analysis for the PAG; these were the dorsomedial periaqueductal gray, dorsolateral periaqueductal gray, and ventrolateral periaqueductal gray (VL-PAG). Lastly, three brain regions were selected for quantitative analysis for the TCC: the superficial layer (spinal trigeminal tract or lamina I/II; Supf C), spinal trigeminal nucleus, and the area around the central commissure.

At each level, four randomly selected sections were assessed, and the average number of Fos-positive cells per hemisection were calculated for each level to investigate the distribution pattern in each nucleus without prior knowledge of the treatment of each animal. All counts were performed by the same experimenters to maintain consistency in application of the criteria used to select Fos-positive cells and to reduce the likelihood of individual variability.

### Data analysis

The average mechanical sensitivity threshold value for each time point in each group [i.e., baseline, sleep deprivation, post sleep deprivation (PSD)] and the count of Fos-positive cells per section in each animal were used for statistical analysis. The significant difference in sensory threshold to mechanical stimuli was determined by Fisher’s least statistical difference test after a repeated measured analysis of variance. Data were reported as medians and 25th to 75th percentiles, with 95% confidence intervals. Because quantitative measurements were performed on an ordinal scale, statistical analyses were conducted using the nonparametric Mann–Whitney U test. A probability of *p* < 0.05 at the two-tailed level was considered to indicate statistical significance. Statistical analyses were performed using the Statistical Package in the Social Sciences software (version 12.0 for Windows; IBM Corp., Armonk, NY, USA).

## Results

### Effects of REM sleep deprivation on sensory threshold to mechanical stimuli

**Fig. 1:**
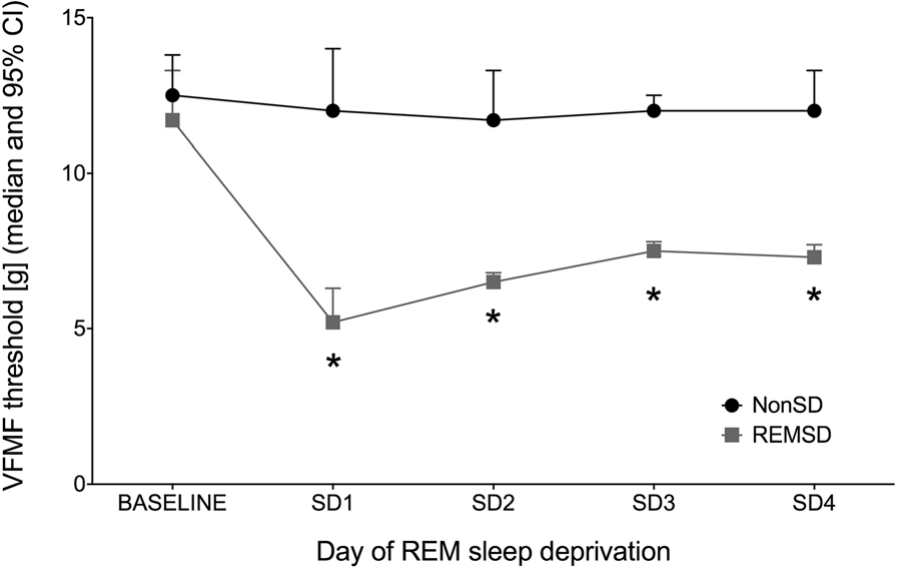
The time course of the VFMF threshold level in each group. The REMSD group showed a significantly decreased VFMF threshold. (* indicates *p* < 0.01 compared to the NonSD group, error bar indicates 95% confidence interval). Abbreviations: SD: days of sleep deprivation; [g]: gram

The VFMF threshold of the REMSD group significantly decreased during the sleep deprivation period versus the baseline. In comparison with the NonSD group, the REMSD group had a significantly lower VFMF threshold after one day of sleep deprivation (*p* = 0.003). This difference lasted for the rest of the sleep deprivation period (Fig. 1).

### Effects of REM sleep deprivation on neuronal FOS immunoreactivity

**Fig. 2:**
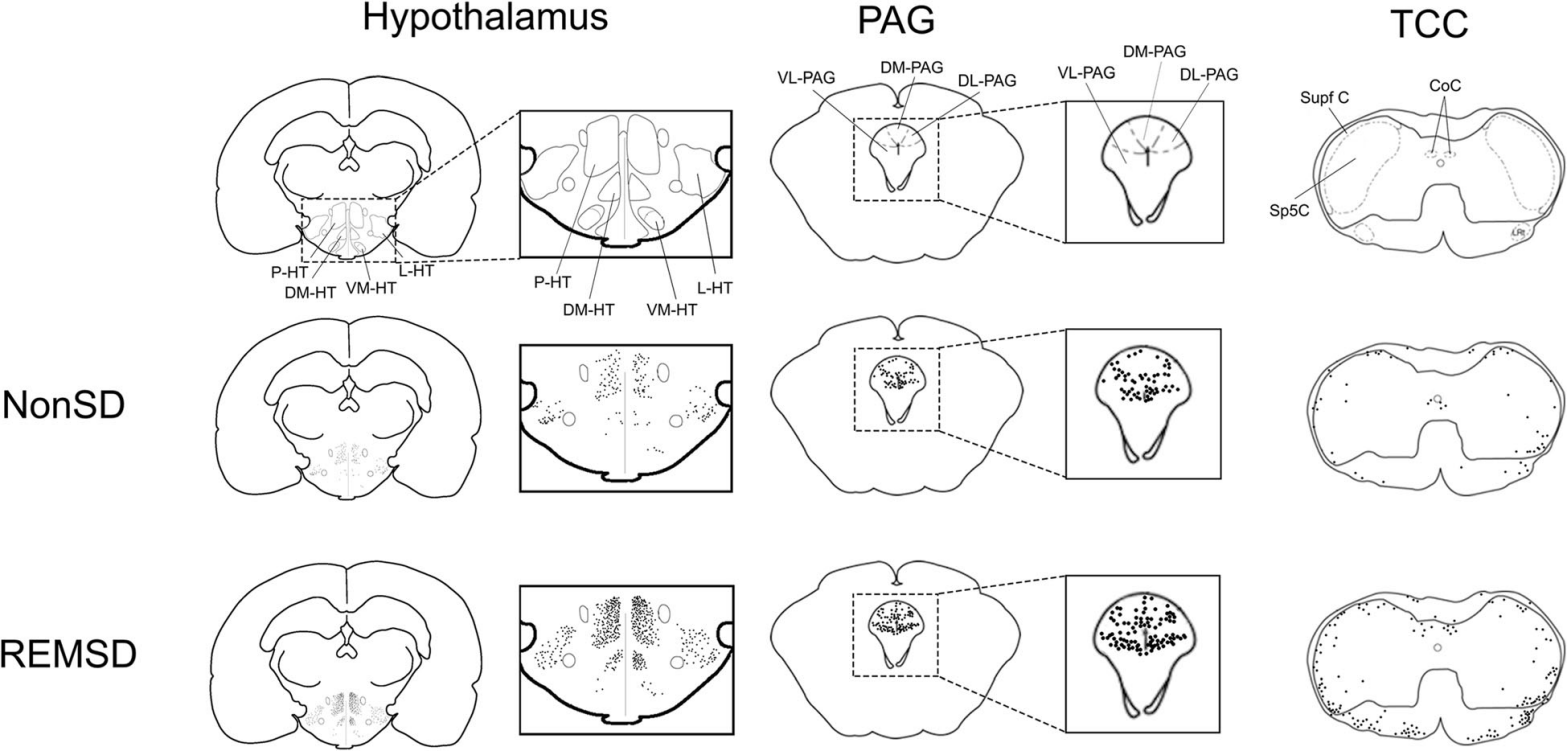
Visual comparisons of the distributions of c-Fos-positive cells in the hypothalamus, PAG, and TCC in each group after REM sleep deprivation. The number of C-Fos-positive cells was significantly higher in the P-HT, DM-HT, VL-PAG, and Supf C. Abbreviations: PAG: periaqueductal gray; TCC: trigeminal nucleus caudalis; P-HT: posterior hypothalamus; DM-HT: dorsomedial hypothalamus; VL-PAG: ventrolateral PAG; Supf C: superficial layer

**Fig. 3:**
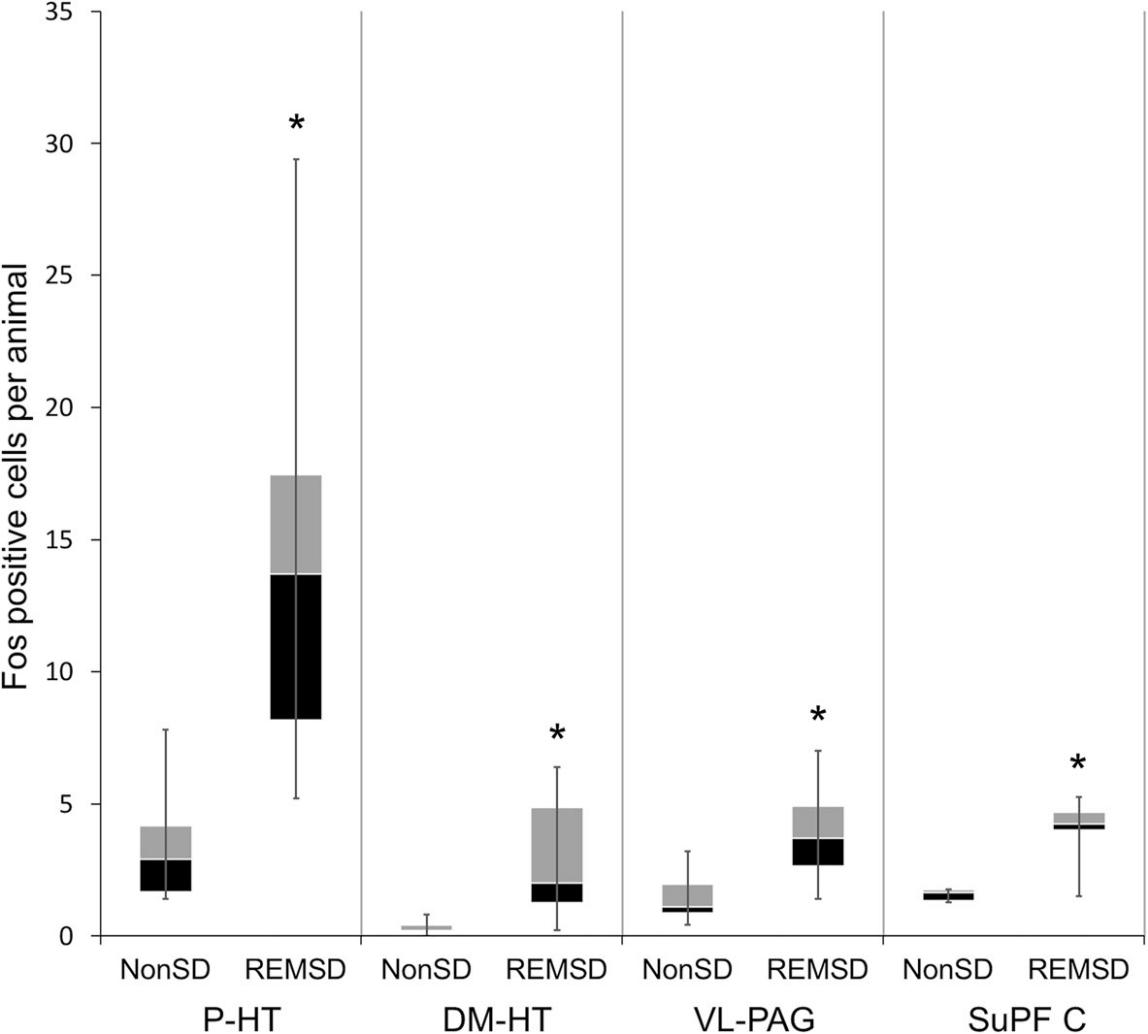
Quantitative comparison of the number of Fos-positive cells per animal in the P-HT, DM-HT, VL-PAG, and superficial layer of the trigeminal nucleus caudalis in each group (* indicates *p* < 0.05). Box plot displaying the median, 25th percentile (lower box), 75th percentile (upper box), and maximum to minimum range (whiskers)

An integrated count of Fos-positive cells for each group is presented in Fig. 2. In the hypothalamus, the number of Fos-positive cells in the P-HT (*p* = 0.010) and DM-HT (*p* = 0.024) in the REMSD group were significantly higher compared to the NonSD group (Table 1, Fig. 3). In the PAG, the number of Fos-positive cells in the VL-PAG was significantly higher in the REMSD group compared to the NonSD group (*p* = 0.016). In the TCC, the number of Fos-positive cells in the Supf C was significantly higher in the REMSD group compared to the NonSD group (*p* = 0.019).

**Table 1:** The median number of Fos-positive cells (25th–75th percentile) per hemisection per animal in both groups

	NonSD	REMSD	*p*-value
Hypothalamus			
P-HT	2.90 (1.65–4.15)	13.70 (6–17.45)	0.010
DM-HT	0.20 (0.20–0.40)	2.00 (1.25–4.85)	0.024
VM-HT	0.10 (0–0.20)	0.20 (0–0.55)	0.607
L-HT	2.20 (1–4)	3.7 (2.85–5.75)	0.146
PAG			
DM-PAG	0.2 (0–0.55)	0.5 (0.05–1.1)	0.318
DL-PAG	0.4 (0.2–1.65)	0.8 (0.4–1.8)	0.326
VL-PAG	1.1 (0.85–1.95)	3.7 (2.65–4.9)	0.016
TCC			
Supf C	1.63 (1.31–1.75)	4.25 (4–4.69)	0.019
Sp5C	0.25 (0.06–0.63)	0.25 (0.06–0.25)	0.735
CoC	0.13 (0–0.25)	0 (0–0.38)	1.000

*Abbreviations: P-HT* posterior hypothalamus, *DM-HT* dorsomedial hypothalamus, *VM-HT* ventromedial hypothalamus, *L-HT* lateral hypothalamus, *PAG* periaqueductal gray, *VL-PAG* ventrolateral PAG, *TCC* trigeminal nucleus caudalis, *Supf C* superficial layer

### Effects of REM sleep deprivation on facial allodynia induced by supradural capsaicin infusion

**Fig. 4:**
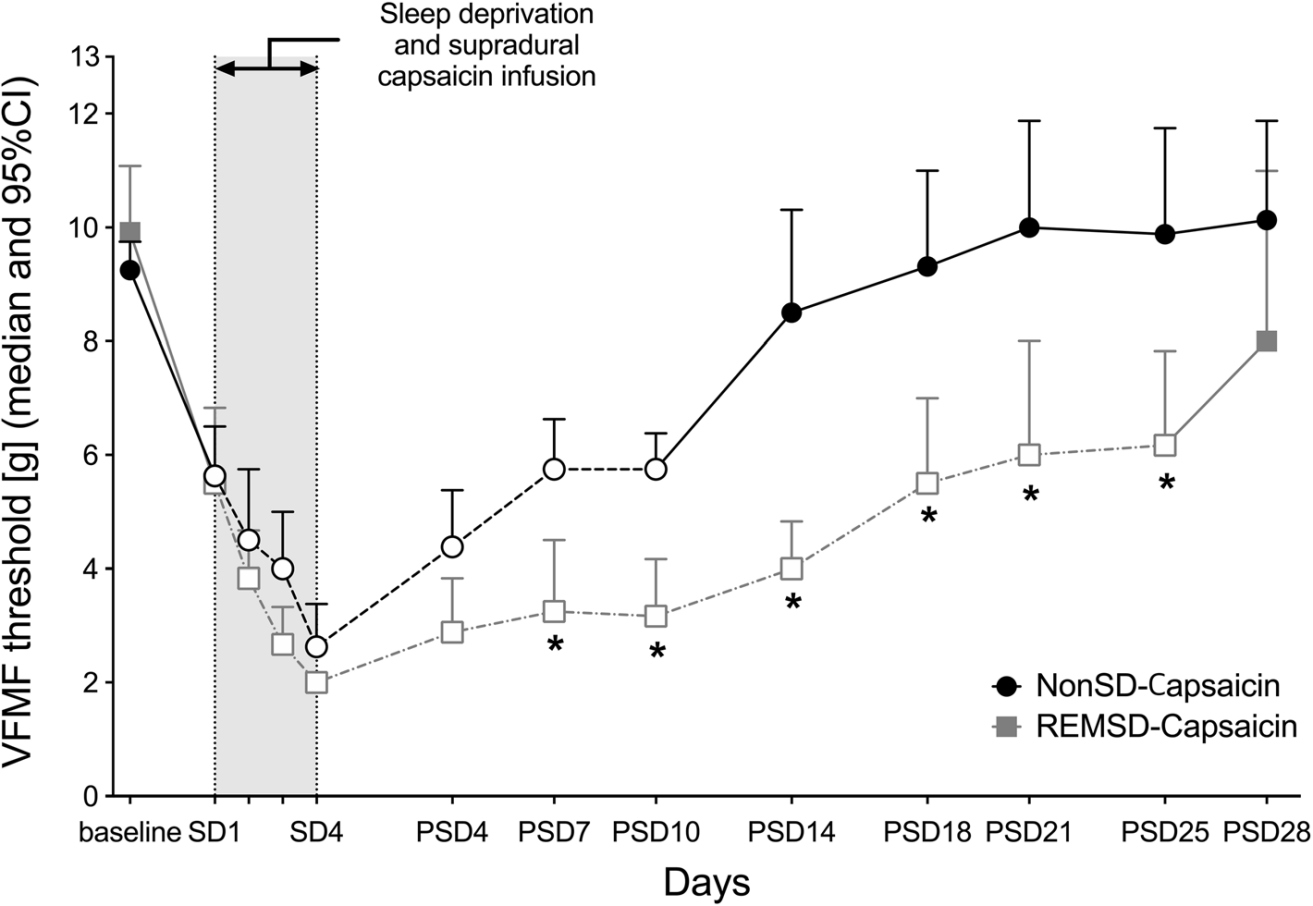
The time course of the VFMF threshold level in each supradural capsaicin infusion group. The duration of reduced VFMF threshold in the REMSD-Capsaicin group was significantly longer than in the NonSD-Capsaicin group (* indicates *p* < 0.05 between groups; blank circles and squares with a dotted-line indicate *p* < 0.05 compared to baseline, error bar indicates 95% confidence interval). Abbreviations: SD: days of sleep deprivation; PSD: days of post-sleep deprivation; [g]: gram

Following supradural capsaicin infusion, the decreased VFMF threshold of the NonSD-Capsaicin group was maintained until Day 10 PSD (*p* = 0.000) and returned to the baseline at Day 14 PSD (*p* = 0.300). In the REMSD-Capsaicin group, the reduced VFMF threshold was maintained until Day 25 PSD (*p* = 0.003) and returned to the baseline at Day 28 PSD (*p* = 0.172). Based on a comparison between the two groups, there were significant differences in VFMF threshold from Day 7 PSD (*p* = 0.014) to Day 25 PSD (*p* = 0.013) (Fig. 4).

## Discussion

The results of the present study can be summarized as follows: (1) REM sleep deprivation induced nociceptive transmission and neuronal activation in pain-related brain areas and (2) REM sleep deprivation prolonged the duration of facial allodynia induced by supradural capsaicin infusion.

Although there are some methodological variations between our study and previous reports, sleep deprivation has previously been found to decrease mechanical sensitivity thresholds, while recovery of sleep restored the thresholds to steady-state basal levels [28–30]. Hick et al. reported that this enhanced nociceptive pain response appeared early in REM sleep deprivation treatment and persisted for 96 h after termination of sleep deprivation [29, 30]. The duration of sleep deprivation was not related to the degree of change in pain threshold. In a study by Onen et al., pain thresholds were decreased at 48 h after REM sleep deprivation but subsequently normalized immediately after recovery of sleep [28]. Vanini et al. studied the effects of total sleep deprivation maintained by mild auditory and tactile stimulation [8] and found that sleep deprivation-related allodynia was diminished at 24 h after sleep recovery.

To focus more on headache pathophysiology, we measured sensory threshold to mechanical stimuli in the periorbital area using the VFMF test in the present study. The decrease in sensory threshold to mechanical stimuli, which corresponds to the facial allodynia of migraine patients, is known to play an important role in migraine pathogenesis [22]. In our investigation, a change in sensitivity to mechanical stimuli appeared after 24 h of sleep deprivation and continued for the remaining 96 h of sleep deprivation. These results may provide a basis for the existence of an association between sleep deprivation and headaches [4, 28, 29]. The mechanism by which sleep deprivation worsens headaches is still unclear. It is known that REM sleep deprivation antagonizes the antinociceptive activity of opioids, which is a suggested mechanism for the effect of sleep deprivation on the central pain modulation system [10]. Interestingly, the mechanisms involving central control of sleep and pain are similar in many ways [31].

The results of the present study—specifically, the neuronal activation in the hypothalamus (P-HT, DM-HT), periaqueductal gray (VL-PAG), and trigeminal nucleus caudalis (Supf C)—suggest that sustained facial allodynia following REM sleep deprivation may be associated with central modulation of nociceptive transmission. The hypothalamus modulates autonomic functions, sleep, and pain [32]. The projections from the basal forebrain to the hypothalamus provide a putative mechanism for the circadian regulation of sleep–wake cycles [33]. In one study, sleep deprivation increased Fos expression in orexin neurons in the hypothalamus in correlation with the amount of sleep [34]. The orexin pathway of the hypothalamus has been found to modulate pain and the sleep cycle [33, 35, 36]. The posterior and dorsomedial nuclei of the hypothalamus have been implicated in descending modulation of pain through their connections with the periaqueductal gray and trigeminal nucleus caudalis [22, 37, 38]. The results associated with deep brain stimulation in the posterior hypothalamus for the treatment of chronic cluster headaches and the analgesic effect of morphine injection to the posterior hypothalamus suggest that the posterior hypothalamus plays a role in headache pain modulation [32, 39]. Stimulation of the dura mater has also resulted in upregulation of Fos immunoreactivity in the posterior and dorsomedial hypothalamic nuclei [38].

Activation of periaqueductal gray modulates nociceptive transmission [12, 37]. The electrical stimulation of PAG may disturb the dorsal raphe nucleus, which is related to pain modulation by a serotonergic pathway [40]. A positron emission tomography (PET) study of migraineurs reported that the periaqueductal gray becomes activated during migraine attacks, which supported the idea that PAG acts as a migraine generator through a change in pain modulation [35]. Electrical stimulation of the ventrolateral PAG significantly inhibits afferent trigeminal nociceptive traffic, and Fos expression in the ventrolateral PAG increased after trigeminovascular stimulation [41, 42]. REM sleep deprivation increases pain independent of its duration or the characteristic of the nociceptive stimulus and decreases morphine analgesia at the periaqueductal gray level [14]. The ventrolateral PAG has been proposed as an important site in the regulation of REM sleep [33, 43]. Additionally, GABAergic input from the ventrolateral PAG decreased during REM sleep, and inactivation of the ventrolateral PAG induced an increase in the amount of REM sleep [44, 45].

The role of the trigeminal nucleus caudalis in the sleep–wake cycle has not been well-investigated to date. The trigeminal nucleus caudalis receives peripheral nociceptive pain from various cranial structures and supraspinal modulation from the brain stem, including the periaqueductal gray and hypothalamus [46]. The results involving neuronal activation in the Supf C after REM sleep deprivation suggest a link between sleep and TCC activation.

We used a supradural capsaicin infusion model for investigating the long-term influence of sleep deprivation on headaches. In the present study, facial allodynia induced by supradural capsaicin infusion was maintained for 10 days when rats were allowed ad libitum sleep, which is consistent with outcomes in previous studies [23, 47]. When rats were placed in a REM sleep deprivation environment, the duration of facial allodynia was prolonged to 25 days. This long-lasting negative impact of REM sleep deprivation on facial allodynia may suggest the potential of REM sleep deprivation to contribute to the development of headache chronicity. The present study supports the results of previous studies that sleep deprivation over a period of time may induce long term effects through molecular, cellular, and network changes [48–50].

The present study has a limitation in that only mechanical stimulation methods were used to evaluate sleep and facial pain. Sleep deprivation may affect cognition and emotion as well as pain. The cognition, emotion, or stress may have some interactions with pain perception. Further research on cognitive and emotional influences on sleep and pain transmission will be helpful. Additionally, a skilled rater who was blinded to the study group assignment performed the experimental procedure, but the validity of data analysis was not double-checked, so there could be a problem associated with inter-rater reliability. The single-platform method was used in the present study for the main purpose of reducing REM sleep, but it also could affect some slow-wave sleep [51, 52]. In a previous study of sleep deprivation with the small-platform method, slow-wave sleep was affected, while REM was completely eliminated [51]. The procedural factors, including immobilization and the single-platform method could be confounding factors related to stress. To minimize these confounding factors, we carefully designed the experiment and provided sufficient habituation periods. Another limitation is that the current experiments did not examine stress responses during the process of sleep deprivation, which can influence various aspects of pain-related behaviors.

## Conclusion

In the present study, we confirmed that REM sleep deprivation increased the sensitivity to mechanical stimuli and neuronal activation in brain areas related to nociceptive transmission. Furthermore, we suggested that REM sleep deprivation may contribute to the chronicity of headaches using an animal facial allodynia model.

## Abbreviations

DM-HT: Dorsomedial hypothalamus
PAG: Periaqueductal gray
PBS: Phosphate-buffered saline
P-HT: Posterior hypothalamus
PSD: Post sleep deprivation
REM: Rapid eye movement
Supf C: Spinal trigeminal tract or lamina I/II
TCC: Trigeminocervical complex
VFMF: Von Frey monofilament
VL-PAG: Ventrolateral periaqueductal gray
VM-HT: Ventromedial hypothalamus
